# Modulation of Short-Chain Fatty Acids as Potential Therapy Method for Type 2 Diabetes Mellitus

**DOI:** 10.1155/2021/6632266

**Published:** 2021-01-04

**Authors:** Ruiqi Tang, Lanjuan Li

**Affiliations:** State Key Laboratory for Diagnosis and Treatment of Infectious Diseases, National Clinical Research Centre for Infectious Diseases, Collaborative Innovation Centre for Diagnosis and Treatment of Infectious Diseases, The First Affiliated Hospital, College of Medicine, Zhejiang University, Hangzhou, Zhejiang 310003, China

## Abstract

In recent years, the relationship between intestinal microbiota (IM) and the pathogenesis of type 2 diabetes mellitus (T2DM) has attracted much attention. The beneficial effects of IM on the metabolic phenotype of the host are often considered to be mediated by short-chain fatty acids (SCFAs), mainly acetate, butyrate, and propionate, the small-molecule metabolites derived from microbial fermentation of indigestible carbohydrates. SCFAs not only have an essential role in intestinal health but might also enter the systemic circulation as signaling molecules affecting the host's metabolism. In this review, we summarize the effects of SCFAs on glucose homeostasis and energy homeostasis and the mechanism through which SCFAs regulate the function of metabolically active organs (brain, liver, adipose tissue, skeletal muscle, and pancreas) and discuss the potential role of modulation of SCFAs as a therapeutic method for T2DM.

## 1. Introduction

The alarmingly high worldwide incidence of type 2 diabetes mellitus (T2DM) and its complications has made it one of the major causes of death. T2DM is a major health issue worldwide. The International Diabetes Federation has estimated that 463 million adults worldwide are living with diabetes currently; 90% of whom have T2DM. This estimate is projected to be 700 million by 2045 [1].

**Figure 1:**
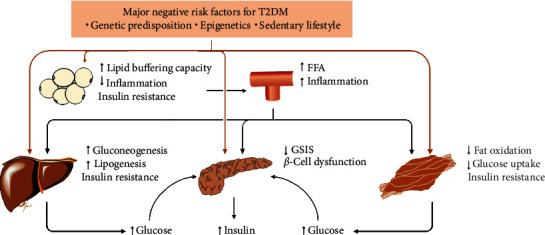
T2DM pathophysiology. A matrix of negative genetic, epigenetic, and lifestyle factors interact with one another and induce dysfunction of pancreatic *β*-cells and insulin resistance in the liver, skeletal muscle, or adipose tissue, thereby leading to the development of hyperinsulinemia and hyperglycemia. Moreover, once reduced lipid-buffering capacity in adipose tissue occurs, circulating lipid concentrations increase, leading to ectopic fat storage in the liver, skeletal muscle, and pancreas as well as the development of insulin resistance and dysfunction of pancreatic *β*-cells. In addition, inflamed adipose tissue results in a low-grade systemic inflammation, which contributes to the development of insulin resistance and T2DM. FFA, free fatty acid; GSIS, glucose-stimulated insulin secretion; T2DM, type 2 diabetes mellitus.

Insulin resistance in insulin-sensitive tissues such as the liver, muscle, and adipose tissue and dysfunction of pancreatic *β*-cells can contribute to the development of hyperglycemia, hyperinsulinemia, insulin resistance, and T2DM [2]. Over the past two decades, information on abnormal signaling by adipocytes and subclinical inflammation that contributes to the prediabetic state has expanded understanding of the complexity of T2DM pathophysiology beyond the classic triumvirate of pancreatic *β*-cells, skeletal muscle, and the liver (Figure 1) [3].

Intestinal microbiota (IM) has a vital role in the modulation of glucose homeostasis and the pathogenesis of metabolic diseases, including T2DM [3, 4]. IM composition is shifted away from species that produce butyrate in people with prediabetes or T2DM compared with that in controls [5, 6]. Insulin sensitivity is improved in obese individuals after receiving transplantation of fecal microbiota from lean donors, which is associated with an increase in the abundance of acetate- or butyrate-producing bacteria [7, 8]. Animal studies support a causal role for IM in the development of obesity, insulin resistance, and T2DM [9, 10]. In addition, alterations in IM have been associated with the development of diabetes-related chronic low-grade inflammation [11, 12].

Clinical trials have indicated that an increase in the intake of nondigestible carbohydrates (dietary fiber) is a possible nutritional strategy to modulate IM, thereby preventing and alleviating the disease phenotypes of T2DM [13–16]. Such dietary fiber supports the growth of symbiotic bacteria. In return, fermentation of these indigestible carbohydrates by these bacteria produces short-chain fatty acids (SCFAs) such as acetate, butyrate, and propionate. The beneficial effects of dietary fiber are often considered to be mediated by SCFAs through the provision of energy sources and reduction of inflammation, as well as regulation of glucose homeostasis and energy homeostasis [4]. The beneficial effect of SCFAs on glucose control, lipolysis, resting-energy expenditure, body weight, and insulin sensitivity has been shown in animals [17–19] and humans [13, 20–22]. SCFAs have been shown to increase insulin sensitivity and promote glucose homeostasis, so modulation of SCFAs could provide a unique approach to T2DM treatment.

**Figure 2:**
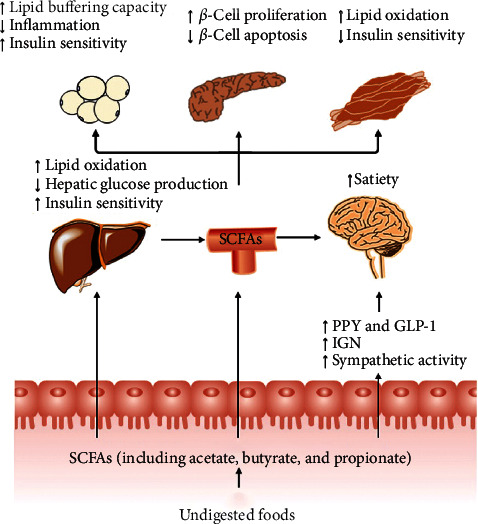
Impact of gut-derived SCFAs in T2DM. SCFAs (acetate, butyrate, and propionate) are produced from the fermentation of indigestible foods in the distal intestine by gut microbiota. In the distal gut, acetate, propionate, and butyrate stimulate the secretion of the “satiety” hormones GLP-1 and PYY in enteroendocrine-L cells, which leads to metabolic benefits upon satiety and glucose homeostasis. Furthermore, butyrate and propionate induce IGN and sympathetic activity, thereby beneficially leading to control of body weight and glucose homeostasis. Very little propionate and butyrate and a high concentration of acetate reach the circulation. They can also affect the metabolism and function of peripheral tissues directly (e.g., liver, adipose tissue, and muscle). Furthermore, circulating levels of acetate and propionate might cross the BBB and regulate satiety *via* CNS-related mechanisms. BBB, blood-brain barrier; CNS, central nervous system; GLP-1, glucagon-like peptide-1; GSIS, glucose-stimulated insulin secretion; IGN, intestinal gluconeogenesis; PYY, peptide YY; SCFAs, short-chain fatty acids; T2DM, type 2 diabetes mellitus.

In this review, we discuss recent studies that provide evidence for the role of microbial SCFAs (acetate, propionate, and butyrate) in T2DM pathogenesis. We provide an overview of the biological properties of SCFAs and their impact on metabolic homeostasis. The effects of SCFAs and nondigestible carbohydrates on the metabolism and function of the gut-brain axis, liver, adipose tissue, skeletal muscle, and pancreas in relation to energy homeostasis, insulin sensitivity, and insulin secretion are also discussed (Figure 2) [23]. Finally, we discuss the potential of SCFAs as novel therapeutics for T2DM.

## 2. Overview of SCFAs

### 2.1. Metabolism and Systemic Concentrations of SCFAs

SCFAs are saturated fatty acids with chain lengths of 1–6 carbon atoms. Due to a lack of the enzymes essential for digestion of dietary fiber in the human gut, SCFAs are the primary metabolites from the fermentation of incompletely hydrolyzed dietary foods by specific gut microbiota in the colon through various pathways (Table 1) [4]. The total concentration of SCFAs in the gut is 0.5–0.6 mol per day [24] depending on the diet, bacterial composition of the gut, and intestinal transit time [25]. Acetate (C2), propionate (C3), and butyrate (C4) are the most abundant SCFAs found in the gut (≥95%) with a molar ratio of roughly 3 : 1 : 1, respectively [3]. The SCFAs produced in the gastrointestinal tract are absorbed rapidly by colonocytes, with only <10% excreted in feces [26]. SCFAs are absorbed by colonocytes mainly through four transport mechanisms: passive diffusion; exchange with bicarbonate; transport by monocarboxylate transporters (MCTs); through sodium-coupled MCT1 [27].

After being absorbed by colonocytes, SCFAs are used as substrates in mitochondrial *β*-oxidation and the citric acid cycle to generate energy [28]. Among SCFAs, butyrate is the primary energy source for colonocytes [4], and propionate is a gluconeogenic substrate [17]. SCFAs that are not metabolized in colonocytes are transported to the liver through the portal circulation, where SCFAs are used as energy substrates for hepatocytes by acetyl-CoA synthetases (ACS) [28]. In addition, in the liver, acetate and butyrate are substrates for the synthesis of cholesterol and long-chain fatty acids [29], and propionate is converted into glucose through the tricarboxylic acid (TCA) cycle [27]. Uptake of propionate and butyrate in the liver is significant, whereas acetate uptake in the liver is negligible [3]. This situation arises because of the low substrate affinity of hepatic mitochondrial ACS1 (which can activate acetate) and the absence of cytosolic ACS2 in the liver, which is present in other organs (e.g., heart and skeletal muscles), where it can be utilized as fuel [28]. SCFAs absorbed in the sigmoid colon and rectum can also reach the systemic circulation directly through the inferior vena cava [23]. Consequently, only 40%, 10%, and 5% of microbial acetate, propionate, and butyrate, respectively, reach the systemic circulation. The plasma concentration (in *μ*M) of acetate, propionate, and butyrate has been estimated to be 19−160, 1–13, and 1–12, respectively [23].

In addition, SCFAs can cross the blood-brain barrier (BBB) *via* MCTs to inform the brain of the intestinal metabolic state [30]. In the brain, acetate is used as an important energy source for astrocytes [25]. The concentration of acetate and propionate in the cerebrospinal fluid of healthy individuals is ∼31 *μ*M and ∼62 *μ*M, respectively [31, 32]. It has been shown that intravenous or colonic infusions of acetate lead to ∼3% and ∼2% acetate taken up by the brain, respectively [33]. However, butyrate uptake in the brain is very low (only 0.006% of the injected dose in primates) [34]. Moreover, no measurable brain uptake of acetate has been detected up to 76 min after intravenous injection in humans [25].

In summary, SCFAs are small-molecule metabolites produced from microbial fermentation of indigestible carbohydrates. Butyrate and propionate are metabolized mainly in the colon and liver, whereas acetate is the main SCFA to enter the circulation. In addition, circulating levels of acetate and propionate can cross the BBB, but uptake of SCFAs in the brain is very low.

### 2.2. Cellular Signaling Pathways of SCFAs

SCFAs are used not only as essential energy sources but also function as signaling molecules because they activate orphan G protein-coupled receptors (GPRs) and inhibit histone deacetylases (HDACs). In this way, they exert several effects to improve metabolic homeostasis and energy homeostasis. The interactions of SCFAs with specific cellular signaling pathways have a potentially key role in SCFAs-mediated regulation of T2DM pathogenesis and are described below.

#### 2.2.1. GPR Activation

**Figure 3:**
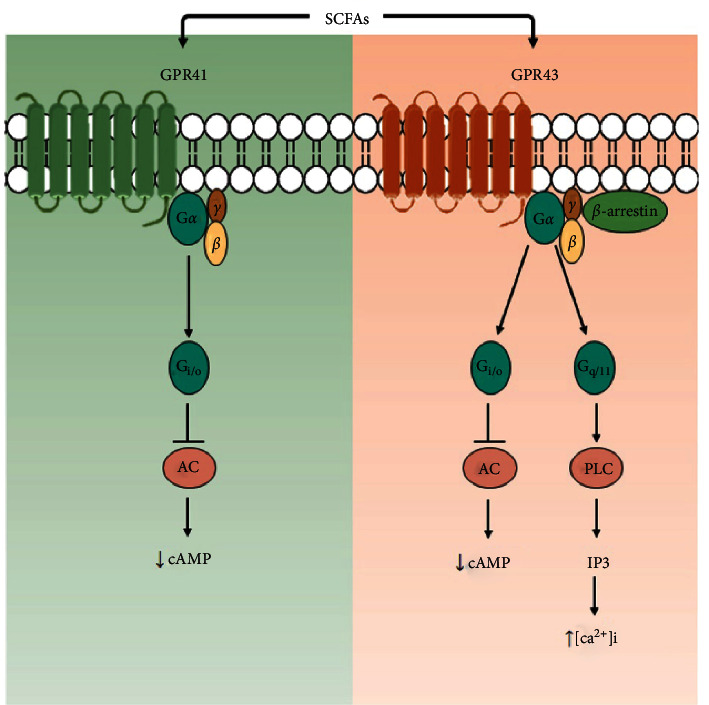
Signaling pathways of GPR41 and GPR43 activated by SCFAs. The signaling pathway downstream of each receptor is illustrated. AC, adenylate cyclase; [Ca^2+^]_i_, intracellular Ca^2+^ concentration; GPR, G protein-coupled receptor; IP3, inositol trisphosphate; PLC, phospholipase C; SCFAs, short-chain fatty acids.

GPR41 and GPR43 are the best-studied SCFA receptors, which have been identified as free fatty acid (FFA) receptor 3 (FFAR3) and FFAR2, respectively [35]. GPRs are seven transmembrane-spanning proteins that detect extracellular molecules and induce intracellular signaling cascades and cellular responses involving different G protein heterotrimers or arrestins [36]. If GPRs are activated by ligands, the G*α* subunit of the heterotrimers (which bears most responsibility for coupling with receptors) disassociates from the G*βγ* subunits and further affects intracellular signaling proteins depending on the type of G*α* subunit (e.g., G*α*_i/o_ and G*α*_q/11_) [37]. GPR41 couples with pertussis toxin-sensitive G*α*_i/o_ proteins. GPR43 couples not only with G*α*_i/o_ but also with the pertussis toxin-insensitive G*α*_q/11_ proteins [38]. Activation of GPR41 and GPR43 by SCFAs *via* G*α*_i/o_ inhibits the activity of adenylate cyclase (AC), which leads to a reduction of cyclic adenosine monophosphate (cAMP) generation. GPR43 activation by SCFAs *via* G*α*_q/11_ activates phospholipase C (PLC), promotes activation of inositol trisphosphate (IP3) receptors located on the endoplasmic reticulum, and leads to Ca^2+^ release from the endoplasmic reticulum (Figure 3) [35]. G*βγ* subunits also activate various molecules, such as the isoform of AC and phospho-inositide-3-kinase. Moreover, *β*-arrestin-2 (a negative regulator of GPR signaling) is recruited by GPR43 activation, which desensitizes GPR signaling *via* G proteins and induces the endocytosis of GPRs [39]. In addition, *β*-arrestin functions as a “scaffold protein” to link GPRs to intracellular signaling pathways and consequently activates the mitogen-activated protein kinase (MAPK) cascade [40].

The potencies of individual SCFAs in activating GPR43 in humans are ordered as *C*2 = *C*3 > *C*4, and those for GPR41 are ordered as *C*3 > *C*4 > *C*2 [35]. GPR43 is expressed in adipose tissue, intestines, pancreatic *β*-cells, and immune tissues [35, 41]. GPR41 is expressed in adipose tissues, intestines, the peripheral nervous system, and immune cells [35, 41]. Thus, GPR43 and GPR41 have important roles in the SCFAs-induced beneficial effects of various physiological functions and systemic glucose homeostasis.

#### 2.2.2. HDAC Inhibition

HDACs are a group of proteases that deacetylate histones and nonhistone proteins, ensuring that they can negatively charge DNA, “curl” chromatin, and inhibit gene transcription. The opposing enzymes, histone acetyltransferases (HATs), transfer the acetyl group of acetyl-CoA to histones, dissociate DNA from histone octamers, relax the nucleosome structure, make transcription factors bind to DNA-binding sites, and activate gene transcription. HATs and HDACs maintain acetylation of histone and nonhistone proteins in dynamic equilibrium to regulate physiological functions, such as inflammation, pancreas development, glucose metabolism, and insulin signaling [24, 42]. However, overexpression and aberrant recruitment of HDACs are associated with T2DM pathogenesis [43].

SCFAs are natural inhibitors of HDACs. SCFAs can act directly on HDACs by entering cells through transporters or act indirectly on HDACs through GPR activation [44]. Evidence highlighting the beneficial effects of SCFA-mediated HDAC inhibition in T2DM has arisen mostly from studies using butyrate. Butyrate has been shown to inhibit HDAC3, suppress peroxisome proliferator-activated receptor (PPAR)-*α* expression, and induce hepatic fibroblast growth factor 21 (*Fgf21*) transcription, which promotes lipid oxidation, triglyceride (TG) clearance, and ketogenesis in the liver [45]. Furthermore, the butyrate-mediated inhibition of HDAC increases nuclear factor erythroid 2-related factor 2 (*Nrf2*) expression *via* the coactivator P300 at the *Nrf2* promoter [46], which has been shown to lead to an increase of its downstream targets to protect against diabetes-induced oxidative stress and inflammation in diabetic mice [47]. In addition, the deacetylase inhibition induced by butyrate also enhances mitochondrial activity [48].

Propionate and acetate can also improve T2DM by inhibiting HDACs [49]. In 3T3-L1 adipocytes, propionate (20 mM) was shown to increase the rate of lipolysis *in vitro* through HDAC inhibitory activity to a similar extent as that by butyrate (5 mM). However, acetate (5 mM) did not affect lipolysis [49]. This may be because of the high mitochondrial and lipogenic demand for two-carbon acetyl units from exogenous acetate in adipocytes, leaving it to contribute to histone acetylation only sparingly [50]. However, acetate can inhibit HDACs in the liver, leading to amelioration of hepatic lipid dysregulation and enhancement of insulin sensitivity in diabetic rats [51] Moreover, acetate released from histone deacetylation can be “recaptured” to supply the acetyl units for HATs [50], indicating a complex role of acetate in histone acetylation.

Altogether, the interaction with GPRs and/or inhibition of HDACs have critical roles in the beneficial effects of SCFAs in T2DM pathogenesis. However, understanding of how SCFAs inhibit HDACs and regulate posttranslational modifications is in its preliminary stages. Future studies should make use of epigenetics and transcriptomics to obtain comprehensive understanding of the part played by SCFAs in T2DM pathogenesis.

## 3. Beneficial Effects of SCFAs on Energy and Glucose Homeostasis

A deficiency of SCFAs has a central role in T2DM development [52]. A metagenome-wide association study of IM in Chinese patients with T2DM showed a moderate degree of intestinal dysbiosis with a lower abundance of butyrate-producing bacteria [5]. Consistently, fecal-metagenome analyses of European women with T2DM have revealed significant depletion of butyrate-producing microbiota, which exhibited a negative correlation with serum levels of insulin, C-peptide, and TG [6].

The production of SCFAs induced by dietary fiber and resistant starch can improve insulin sensitivity and glucose homeostasis in humans. Supplementation with high amylose-resistant starch has been shown to reduce body fat, increase levels of acetate, early-phase insulin, and glucagon-like peptide- (GLP-) 1, and increase the number of gut microbes that produced acetate in volunteers with normal body weight [53]. Rye-based bread supplemented with resistant starch type 2 increased insulin sensitivity, fasting levels of peptide YY (PYY), GLP-2, acetate, butyrate, and total SCFAs in healthy middle-aged individuals [54]. Furthermore, administration of dietary fiber in T2DM patients enhanced a group of SCFA producers and improved glycated hemoglobin levels, partly *via* increased GLP-1 production, which resulted in T2DM alleviation [13].

Direct administration of SCFAs can influence the homeostasis of glucose metabolism and optimize insulin sensitivity. Acute oral administration of sodium propionate increased resting-energy expenditure and was accompanied by increased whole-body lipid oxidation, in fasted healthy volunteers [21]. Rectal administration of SCFAs mixtures for four days increased fasting fat oxidation, energy expenditure, and plasma levels of PYY and decreased fasting free-glycerol concentrations in normoglycemic overweight men [20].

Overall, the clinical data stated above suggest that modulation of SCFAs could prevent or alleviate T2DM. Nonetheless, those are preliminary results from small-sample studies on the effects of SCFAs on the host's metabolism. More prospective studies involving much larger and more diverse sample sets are needed to investigate further the effects of long-term administration (through different modes of administration) of SCFAs on T2DM.

### 3.1. SCFAs Regulate the Brain's Control of Energy Homeostasis

SCFAs derived from the gut can positively influence the effect of the brain in controlling energy homeostasis and glucose homeostasis. These include reduced energy intake, body weight, hepatic glucose production, and improved insulin sensitivity, all of which reduce T2DM. SCFAs affect the gut-brain axis by regulation of secretion of metabolic hormones, induction of intestinal gluconeogenesis (IGN), stimulation of vagal afferent neurons, and regulation of the central nervous system (CNS) [25].

#### 3.1.1. Regulation of Secretion of Metabolic Hormones

Modulation of the hormones associated with satiety is one of the best-studied mechanisms by which SCFAs regulate appetite and energy intake. Studies [13, 55, 56] have shown that plasma levels of GLP-1 and PYY in overweight adults are increased after acute rectal infusion of sodium acetate [57, 58] or SCFAs mixtures [20] or an oral insulin-propionate ester [59]. SCFAs can trigger the secretion of GLP-1 and PYY from enteroendocrine-L cells [60–64] through GPR41 and GPR43 and/or GPRs-independent signaling by being metabolized to adenosine triphosphate (ATP) as a colonocyte energy source [65]. Production of these gut hormones leads to activation of appetite- and food intake-related brain activity *via* humoral and neural pathways [25]. GLP-1 is an anorexigenic incretin hormone that enhances glucose-dependent insulin secretion [66]. The interaction between circulating levels of GLP-1 and food reward-related central nervous activity in the dorsolateral prefrontal cortex can achieve body weight loss in obese individuals [67]. PYY is costored and cosecreted with GLP-1 by enteroendocrine-L cells [35]. PYY is another anorexic neuropeptide that has been shown to inhibit gastrointestinal movement, suppress appetite, and improve the survival and function of pancreatic *β*-cells, with obvious benefits for T2DM [68].

Moreover, SCFAs can affect the secretion of other metabolic hormones, including leptin and ghrelin. The BBB and vagus nerve are implicated in the effect of these hormones on the brain [69, 70]. Leptin is an anorexic hormone secreted from adipose cells and activates hypothalamic proopiomelanocortin neurons to inhibit food intake [71]. However, the effect of SCFAs on the regulation of leptin production seems controversial [69]. *In vitro* studies have demonstrated consistently that SCFAs stimulate leptin secretion in adipocytes through GPR41 activation. *In vivo* studies have shown that body fat (rather than SCFAs) is the main driver for leptin synthesis [69]. Ghrelin is the main “hunger” hormone. It is produced by ghrelin cells in the stomach and duodenum and activates hypothalamic somatostatin neurons to promote feeding [70]. Acute increases in levels of colonic-derived SCFAs by ingestion of inulin reduce ghrelin levels in lean and obese individuals [72]. However, chronic intragastric infusion of acetate has been found to activate the vagal nervous system and, in turn, stimulate ghrelin secretion in rats, which may promote hyperphagia and metabolic syndrome [73].

Hence, SCFAs may exert beneficial effects on appetite suppression and lower energy intake mainly by regulation of metabolic hormones such as GLP-1, PYY, leptin, and ghrelin. Further investigations on the direct impact and underlying mechanism of action of SCFAs on these hormones are needed to clarify the mechanism through which SCFAs affect energy homeostasis.

#### 3.1.2. IGN Induction

IGN is a brain signal derived from the intestine that plays an important part in glucose homeostasis and energy homeostasis [74]. IGN is induced during the postabsorptive period [17, 74]. It can induce beneficial effects on metabolism, such as a decrease in food intake, acquisition of a food preference, rapid-phase secretion of insulin, and reduction of hepatic glucose production, *via* gut-brain glucose signaling [75]. Because of intense glycolysis in the intestine, physiological portal hypoglycemia occurs during the postabsorptive period. This signals to the brain *via* sodium-coupled glucose cotransporter 3 in the hepatic-portal area (a key link in the portal glucose-sensing process) and promotes the reonset of hunger [74, 75]. IGN activation counterbalances the lowering of portal-area glucose, resulting in hunger inhibition. Interestingly, the hunger-curbing effect of the portal glucose signal induced by IGN involves activation of afferents from the spinal cord and specific neurons in the parabrachial nucleus, rather than the afferents from the vagal nerves [74, 76].

The SCFAs butyrate and propionate activate IGN *via* complementary metabolic processes [17, 77]. Butyrate-induced activation of IGN is mediated by an increase in ATP, which increases intracellular cAMP, but not *via* G*α*_i/o_- or G*α*_q/11_-mediated mechanisms [17]. Propionate (itself a gluconeogenic substrate) activates GPR41 in periportal nerves. It stimulates a gut-brain neural circuit that induces IGN by promoting the local release of vasoactive intestinal peptides [77] and upregulation of methylmalonyl-CoA mutase (the key enzyme in propionate metabolization) [17]. The position of SCFAs upstream of IGN-mediated gut-brain glucose signaling indicates that this function can activate the portal nervous system and its related benefits.

#### 3.1.3. Stimulation of Vagal Afferent Neurons

SCFAs can suppress energy intake by stimulating vagal afferent neurons directly. Oral (but not intravenous) administration of butyrate reduces food intake by activating the gut-brain neural circuit, resulting in inhibition of orexigenic neuropeptide Y neurons in the hypothalamus and neurons within the tractus solitarius and dorsal vagal nuclei [19]. Furthermore, intraperitoneal administration of three SCFAs was shown to reduce food intake by activating vagal afferents in fasted mice in the order *C*4 > *C*3 > *C*2 [78]. This effect was attenuated by systemic capsaicin treatment and hepatic-branch vagotomy that desensitized vagal afferents. Moreover, butyrate-induced sympathetic activity increased phosphorylation of extracellular signal-regulated kinase-1/2 and intracellular Ca^2+^ concentration ([Ca^2+^]_i_) signaling in nodose ganglion neurons [78]; this could have been mediated by GPR41 activation in nodose ganglion neurons [79]. Moreover, the gut-brain neural circuit induced by SCFAs may also promote fat oxidation by activating brown adipose tissue [19]. Surprisingly, chronic intragastric infusion of acetate activated vagal nervous and stimulated ghrelin secretion in rats, which increased caloric intake and weight gain [73]. A recent study revealed that activation of the right (but not the left) upper-gut vagal sensory ganglion stimulated the parabrachio-nigral pathway (which regulates food consumption) in mice [80]. Thus, investigating whether these asymmetric gut-brain pathways of vagal origin might be a mechanism that mediates the differential effects of SCFAs on vagal activity would be worthwhile.

#### 3.1.4. CNS Regulation

Only a few rodent studies have demonstrated that SCFAs derived from the colon can cross the BBB directly and affect the CNS, which are related to appetite and energy intake. Intracerebroventricular injections of acetate were shown to reduce food intake significantly at 1-2 h after injection [33]. In the hypothalamus, acetate is oxidized in the TCA cycle, leading to inactivation of AMP-activated protein kinase (AMPK) and simultaneous inhibition of acetyl-CoA carboxylase (ACC). This action stimulates proopiomelanocortin neurons and suppresses agouti-related peptide neurons, thereby inducing appetite inhibition [33]. Importantly, due to the invasive nature of the studies on the effects of SCFAs on brain function, those studies were limited to *in vitro* and animal studies. Whether colonic-derived SCFAs have a similar role in the human CNS merits further investigation.

In summary, the studies mentioned above provide evidence for the therapeutic benefit of SCFAs on energy homeostasis *via* regulation of appetite-regulating hormones and sympathetic activity. In addition, butyrate and propionate might promote metabolic benefits on glucose homeostasis and body weight *via* induction of IGN, and acetate might directly induce appetite inhibition *via* a central mechanism in the CNS.

### 3.2. SCFAs Induce Preservation of Hepatic Metabolic Function

The gut-liver axis is involved in the beneficial effect of SCFAs on T2DM chiefly by preserving the metabolic function of the liver, including decreasing hepatic glucose production [81] and lipid accumulation [82], modulating hepatic mitochondrial function, and increasing glucose uptake and glycogen synthesis in hepatocytes [83].

SCFAs sustain hepatic metabolic function and insulin sensitivity mainly *via* an AMPK-dependent pathway [84]. AMPK is a necessary regulator for maintaining the homeostasis of the metabolism of energy, glucose, and lipids in the liver. AMPK and its downstream fatty acid oxidation genes increased by acetate administration alleviate hepatic lipid accumulation in mice suffering from nonalcoholic steatohepatitis [85]. Butyrate-induced AMPK increases levels of PPAR coactivator (PGC)-1*α* or ACC in insulin-resistant hepatocytes and in mice [18, 86], which modulate the mitochondrial functions and increased use of substrates (especially fatty acids), leading to reduction of intracellular lipid accumulation. Moreover, propionate activates AMPK in human HepG2 hepatocytes, resulting in downregulation of expression of the gluconeogenesis-related genes glucose-6-phosphatase and phosphoenolpyruvate carboxykinase [81]. Importantly, knockdown of GPR43 expression prevents propionate-induced phosphorylation of AMPK [81]. Activation of GPR43 by SCFAs induces expression of G*α*_i/o_ and G*α*_q/11_, as well as recruitment of *β*-arrestin-2. Although G*α*_i/o_ reduces the production of cAMP from ATP, which can inhibit AMPK activation, the G*α*_q/11_-induced increase in [Ca^2+^]_i_ activates Ca^2+^/calmodulin-dependent protein kinase *β*-dependent phosphorylation of AMPK [81]. Moreover, a recent study showed that *β*-arrestin-2 also contributes to the GPR43-induced activation of AMPK [86]. In summary, SCFAs-induced activation of AMPK can be attributed to GPR43-induced G*α*_q/11_ activation and *β*-arrestin-2 recruitment.

In addition to AMPK activation, SCFAs-induced activation of GPR43 can promote glucose uptake and glycogen metabolism in the liver. In db/db mice and HepG2 cells, butyrate administration was shown to upregulate expression of two glucose transporters and inhibit protein kinase B (Akt) expression which, in turn, activated glycogen synthase kinase 3. This process increased glycogen storage significantly in mice and HepG2 cells [83].

Furthermore, as a broad-spectrum HDAC inhibitor, butyrate also exhibits beneficial effects in the liver by an epigenetic mechanism involving HDAC inhibition. The inhibition of HDAC2 induced by butyrate upregulates expression of hepatic GLP-1R and subsequently promotes GLP-1-dependent activation of insulin pathways. Subsequently, this action stimulates lipid oxidation and improves hepatic steatosis and insulin sensitivity [87]. Butyrate inhibits HDAC3 and HDAC4 significantly, accompanied by an increase in the number of genes participating in *β*-oxidation of fatty acids, which promotes the biogenesis and function of mitochondria in insulin-resistant hepatocytes [86]. In addition, butyrate induces PPAR*α* activation with enhanced histone H3 acetyl K9 (H3K9Ac) modification on its promoter by HDAC1 inhibition, which leads to upregulation of *Fgf21* expression, and enhanced fatty acid oxidation [45, 88].

Taken together, the results stated above suggest that colonic-derived SCFAs might indirectly affect liver function and metabolism by interacting with GPR43 and inhibiting HDAC. These actions might affect hepatic glucose and glycogen metabolism, fatty acid oxidation, and mitochondrial function, with activation of AMPK, Akt, and PPAR*α* being mediators of these effects. Results from *in vitro* and animal studies seem promising, but there is a dearth of clinical research and very little integration of human and animal studies. Thus, it is necessary to investigate the mechanism through which SCFAs affect metabolic function in human livers.

### 3.3. SCFAs Improve Dysfunction in Adipose Tissue

Adipose tissue is the most abundant energy store (in the form of TG) in the human body. Adipose tissue is a lipid-buffering mass that increases plasma TG clearance and inhibits the release of fatty acids into the circulation [23]. If energy expenditure is lower than energy intake, the TG stored in adipose tissue and the rate of lipolysis increase. These actions lead to an overflow of lipids, accumulation of lipids in other peripheral tissues, and adipose tissue inflammation, which contribute to T2DM development. SCFAs can regulate lipid metabolism and reduce inflammation [89] in adipose tissue.

SCFAs can alter lipid metabolism in adipose tissue by promoting lipolysis and inhibiting lipogenesis, with activation of AMPK and *β*3-adrenergic receptors (AR*β*3) in mice [90, 91], pigs [92], and rabbits [93, 94]. Clinical data have suggested the metabolic effects of SCFAs on the dysfunction of adipose tissue, as indicated by decreased free-glycerol concentrations [20] or eliminated FFA in plasma [95]. Acetate induces upregulation of lipolysis-related factors [93, 94], which may due to activation of the GPR43-AMPK pathway [81, 86]. Butyrate administration also activates AR*β*3-mediated lipolysis in adipose tissue by enhancing acetylation of lysine 9 on histone H3 of the AR*β3* promoter [91].

Furthermore, SCFAs can promote adipogenesis in rabbit adipocytes [93, 94] and 3T3-L1 adipocytes *in vitro* [96]. Treatment with acetate, propionate, or butyrate accelerated the differentiation of 3T3-L1 adipocytes by upregulating expression of the enzymes of fatty acid metabolism, including lipoprotein lipase, adipocyte fatty acid-binding protein 4, fatty acid transporter protein 4, and fatty acid synthase (FAS) [96]. GPR41/43-mediated MAPK signaling may be involved in SCFA-induced adipocyte differentiation in rabbits by upregulating downstream adipocyte-specific transcription factors, including PPAR*γ* and differentiation-dependent factor 1 [93, 94]. Nevertheless, clinical studies suggest that SCFAs are not correlated with adipocyte differentiation. Acetate and propionate do not affect the differentiation of human preadipocytes [97]. More evidence is needed to clarify the effects of SCFAs on human adipogenesis. However, propionate inhibits adipogenic differentiation of human chorion-derived mesenchymal stem cells (cMSCs), which is elicited by silencing of GPR43 expression [98]. Since almost all of the tissues contain varying proportions of MSCs, inhibiting the adipogenic differentiation of MSCs with SCFAs may be a way to inhibit the undesirable formation of adipocytes throughout the body.

Few studies have evaluated the direct effect of SCFAs on adipose tissue inflammation. Adipose tissue inflammation plays a part in the development of insulin resistance and T2DM [3, 23]. Macrophages are the most studied of the adipose-derived immune populations. They are believed to be major sources of inflammatory cytokines in response to a high-fat diet (HFD) and obesity [99]. Butyrate reduces macrophage infiltration in adipose tissue in mice, which results in the improvement of insulin sensitivity [100]. Moreover, *in vitro* studies have revealed that GPR41/43-induced activation of G*α*_i/o_ protein is involved in the way propionate reduces tumor necrosis factor-*α* release in macrophages [89]. Those data suggest that SCFAs might counteract adipose tissue inflammation directly.

In summary, SCFAs can increase the lipid-buffering capacity of adipose tissue by promoting lipolysis, inhibiting lipogenesis, and promoting adipogenesis of adipose precursor cells, but inhibiting adipogenic differentiation of cMSCs (which have been identified in numerous tissues). SCFAs may prevent chronic low-grade inflammation by reducing macrophage infiltration in adipose tissue. Most evidence has been obtained from *in vitro* studies of adipocytes derived from animals, which cannot directly reflect the status of SCFAs in human adipocytes. Hence, future research should focus on the metabolic function of SCFAs in humans and human-cell models.

### 3.4. SCFAs Enhance Insulin Sensitivity in Skeletal Muscle

In addition to inhibition of ectopic fat storage by reducing circulating lipid concentrations, SCFAs might also contribute to improvement in skeletal muscle insulin sensitivity by decreasing fatty acid synthesis and increasing lipolysis in skeletal muscle. SCFAs can increase the lipid-oxidation capacity of skeletal muscle by improving mitochondrial function [101, 102]. Mitochondria are essential for maintaining energy homeostasis in skeletal muscle by adaptive reprogramming to meet the demands imposed by an increased lipid supply [103]. Supplementation with butyrate enhances mitochondrial biogenesis in skeletal muscles as indicated by upregulation of expression of most mitochondrial DNA-encoded genes. This action may be involved in GPR41/43 and PGC-1*α* pathways [101]. The butyrate-mediated activity of HDAC inhibitors may also induce nucleosome positioning, which is associated with improving *β*-oxidation and insulin sensitivity [102]. Moreover, SCFAs can decrease fatty acid synthesis by downregulating mRNA expression of FAS, ACC, and PPAR*σ* in longissimus dorsi [92]. However, studies on the effects of SCFAs on the metabolic function of human skeletal muscle are lacking. Therefore, future research should pay attention to SCFAs uptake in skeletal muscle as well as their effect and mechanism of action on oxidative metabolism in human muscle.

### 3.5. SCFAs Regulate Pancreatic Function

Besides the indirect effect of SCFAs on insulin secretion *via* the parasympathetic nervous system and regulation of circulating lipid concentrations, studies have suggested the direct effect of SCFAs on pancreatic *β*-cells. *In vitro* [104] and animal [105, 106] studies revealed that propionate and butyrate inhibited the apoptosis of pancreatic *β*-cells and promoted their proliferation, which led to an increase in pancreatic *β*-cell mass and improved glucose homeostasis. This effect may be related to the SCFAs-mediated HDAC inhibitory activity inducing activation of the MAPK pathway [106], and inhibition of the endoplasmic reticulum stress-related protein kinase R-like ER kinase (PERK)-CCAAT/enhancer-binding protein homologous protein (CHOP) pathway [105]. The MAPK pathway has pivotal roles in the proliferation and differentiation of pancreatic *β*-cells [106] and PERK-CHOP pathway has an important role in the apoptosis of pancreatic *β*-cells [105].

Moreover, SCFAs might influence pancreatic function by regulating insulin secretion. Dietary supplementation with propionate has been shown to increase glucose-stimulated insulin secretion (GSIS) in humans, an effect that is dependent upon G*α*_q/11_-mediated signaling consequent to GPR43 activation [104]. However, propionate inhibited glucose-dependent insulin secretion, which occurred through a G*α*_i/o_ pathway [107]. Interestingly, butyrate supplementation reduced insulin secretion at a basal condition (2.8 mM glucose) but increased GSIS (16.7 mM glucose) released by pancreatic *β*-cells isolated from HFD mice [82]. Acetate has been shown to strongly reduce plasma insulin levels and GSIS from isolated perfused pancreas tissue from rats [108]. The discrepancies mentioned above may be related to the ability of SCFAs to activate GPR41 and GPR43. Studies on GPR41-or GPR43-knockout mice have found that GPR41 and GPR43 are involved in the insulin secretion activity of pancreatic *β*-cells [109, 110]. The SCFAs induced GPR41 to activate G*α*_i/o_ signaling pathways, which reduced cAMP levels in pancreatic *β*-cells and led to reduced insulin secretion from pancreatic *β*-cells [111]. Inconsistently, the SCFAs-induced activation of GSIS was attributed to GPR43-and G*α*_q/11_-dependent actions, which increased [Ca^2+^]_i_ and induced insulin secretion [109, 112, 113]. Thus, SCFAs modify the balance between GPR41 and GPR43 signaling in pancreatic *β*-cells and, therefore, may “fine-tune” insulin secretion to maintain metabolic homeostasis.

The data mentioned above illustrate the ability of SCFAs to regulate pancreatic function and glucose homeostasis. The signaling induced by SCFA-mediated HDACs inhibition contributes to the protection of pancreatic *β*-cells by inhibiting their apoptosis and promoting their proliferation. In addition, SCFAs might regulate insulin secretion through GPRs pathways. However, the physiological importance of the GPR-based dual-coupled signaling mechanism in insulin secretion is not fully understood. Therefore, more studies are needed for further investigation of the mechanism of the effects of the SCFA-GPR axis on the control of insulin secretion and functioning of pancreatic *β*-cells.

## 4. Conclusions and Perspectives

A wide range of preclinical evidence strongly suggests that an increase in SCFAs could be a potential therapeutic method to prevent and/or alleviate T2DM. Evidence in humans is circumstantial, but clinical data indicate the possibility of SCFAs as novel therapeutic agents for T2DM. Observational and intervention studies provide evidence that SCFAs might induce appetite inhibition and affect energy homeostasis by regulating the secretion of appetite-regulating hormones, inducing IGN, stimulating sympathetic activity, and regulating CNS. SCFAs might regulate glucose homeostasis by decreasing glucose production, increasing glucose uptake and glycogen synthesis in the liver, increasing pancreatic *β*-cell mass, and regulating insulin secretion. Furthermore, SCFAs might improve lipid metabolism by increasing the lipid-buffering capacity of adipose tissue and reduce inflammation in adipose tissue, as well as enhancing fatty acid oxidation and mitochondrial function in the liver and skeletal muscle.

Clinical studies have indicated a causal role for SCFAs in metabolic health. However, the metabolic consequences of direct administration of SCFAs in humans are incompletely understood. Clinical trials are needed to verify these effects on humans. Due to the instability of the SCFAs dose delivered to the target, a novel targeting method for colonic delivery of SCFAs should be developed to achieve more consistent and reliable dosing. The gut-host signal axis may be more resistant to such intervention by microbial SCFAs (especially in the insulin-resistant phenotype), so this method should be tested for ≥3 months. In addition, due to interindividual variability in microbiota and metabolism, factors that may directly affect host substrate and energy metabolism, such as diet and physical activity, should be standardized or at least assessed. Moreover, advanced metabolomics, epigenetics, metatranscriptomics, and metagenomics approaches may provide insight into the impact of SCFAs on maintaining insulin sensitivity and metabolic homeostasis in humans. These emerging technologies may offer great potential for the eventual therapeutic translation of SCFAs in T2DM.

## Figures and Tables

**Table 1:** Precursors, biosynthetic pathways, and producers of SCFAs.

SCFAs	Precursors	Pathways	Producers
Acetate	Pyruvate	Acetyl-CoA pathway	Most intestinal bacteria, such as *Bacteroides* spp., *Prevotella* spp., *Ruminococcus* spp., *Bifidobacterium* spp., and *Akkermansia muciniphila*
		Wood–Ljungdahl pathway	*Clostridium* spp., *Streptococcus* spp., and *Blautia hydrogenotrophica*
Propionate	Phosphoenol-pyruvate	Succinic pathway	*Bacteroides* spp., *Dialister* spp., *Phascolarctobacterium succinatutens*, and *Veillonella* spp.
		Acrylic pathway	*Coprococcus catus* and *Megasphaera elsdenii*
	Deoxyhexose	Propanediol pathway	*Ruminococcus obeum*, *Roseburia inulinivorans*, and *Salmonella* spp.
Butyrate	Acetyl-CoA	Acetate CoA-transferase pathway	*Faecalibacterium prausnitzii*, *Eubacterium hallii*, and *Roseburia* spp.
		Butyrate kinase pathway	*Coprococcus catus* and *Coprococcus comes*
	Proteins	Lysine pathway	*Odoribacter splanchnicus* and *Alistipes putredinis*
