# Corrigendum to “Modulation of Short-Chain Fatty Acids as Potential Therapy Method for Type 2 Diabetes Mellitus”

**DOI:** 10.1155/2021/9756586

**Published:** 2021-06-04

**Authors:** Ruiqi Tang, Lanjuan Li

**Affiliations:** State Key Laboratory for Diagnosis and Treatment of Infectious Diseases, National Clinical Research Centre for Infectious Diseases, Collaborative Innovation Centre for Diagnosis and Treatment of Infectious Diseases, The First Affiliated Hospital, College of Medicine, Zhejiang University, Hangzhou, Zhejiang 310003, China

In the article titled “Modulation of Short-Chain Fatty Acids as Potential Therapy Method for Type 2 Diabetes Mellitus” [1], errors were identified in Figures 1 and 2 which were introduced during the preparation of the manuscript. The arrows for adipose tissue in Figure 1 should be pointing down for lipid buffering capacity and pointing up for inflammation. In Figure 2, the arrows for skeletal muscle should be pointing up. The authors confirm that this does not affect the results and conclusion of the article, and the revised figures are included below.

## Figures and Tables

**Figure 1:**
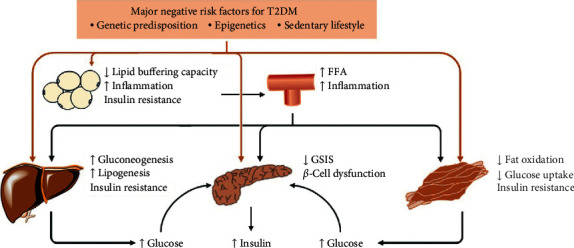
T2DM pathophysiology. A matrix of negative genetic, epigenetic, and lifestyle factors interact with one another and induce dysfunction of pancreatic *β*-cells and insulin resistance in the liver, skeletal muscle, or adipose tissue, thereby leading to the development of hyperinsulinemia and hyperglycemia. Moreover, once reduced lipid-buffering capacity in adipose tissue occurs, circulating lipid concentrations increase, leading to ectopic fat storage in the liver, skeletal muscle, and pancreas as well as the development of insulin resistance and dysfunction of pancreatic *β*-cells. In addition, inflamed adipose tissue results in a low-grade systemic inflammation, which contributes to the development of insulin resistance and T2DM. FFA, free fatty acid; GSIS, glucose-stimulated insulin secretion; T2DM, type 2 diabetes mellitus.

**Figure 2:**
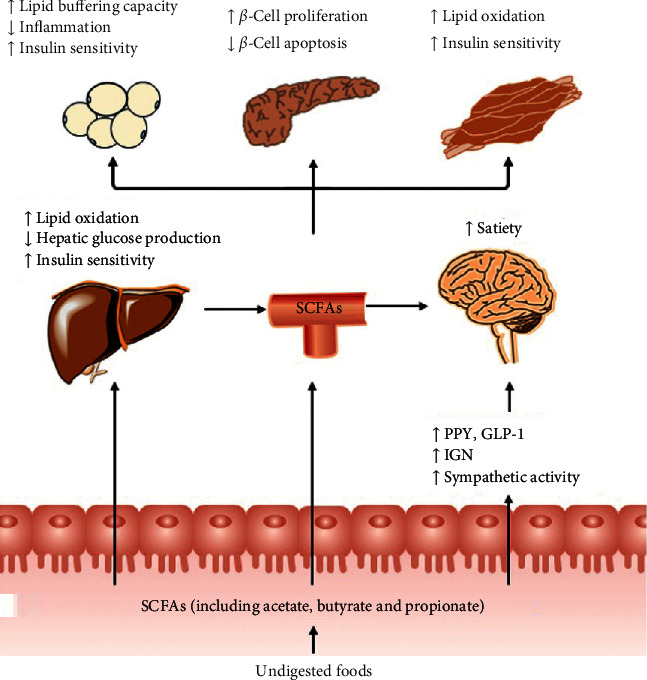
Impact of gut-derived SCFAs in T2DM. SCFAs (acetate, butyrate, and propionate) are produced from the fermentation of indigestible foods in the distal intestine by gut microbiota. In the distal gut, acetate, propionate, and butyrate stimulate the secretion of the “satiety” hormones GLP-1 and PYY in enteroendocrine-L cells, which leads to metabolic benefits upon satiety and glucose homeostasis. Furthermore, butyrate and propionate induce IGN and sympathetic activity, thereby beneficially leading to control of body weight and glucose homeostasis. Very little propionate and butyrate and a high concentration of acetate reach the circulation. They can also affect the metabolism and function of peripheral tissues directly (e.g., liver, adipose tissue, and muscle). Furthermore, circulating levels of acetate and propionate might cross the BBB and regulate satiety *via* CNS-related mechanisms. BBB, blood-brain barrier; CNS, central nervous system; GLP-1, glucagon-like peptide-1; GSIS, glucose-stimulated insulin secretion; IGN, intestinal gluconeogenesis; PYY, peptide YY; SCFAs, short-chain fatty acids; T2DM, type 2 diabetes mellitus.
